# Posterior tibial slope influences joint mechanics and soft tissue loading after total knee arthroplasty

**DOI:** 10.3389/fbioe.2024.1352794

**Published:** 2024-04-15

**Authors:** Ning Guo, Colin R. Smith, Pascal Schütz, Adam Trepczynski, Philippe Moewis, Philipp Damm, Allan Maas, Thomas M. Grupp, William R. Taylor, Seyyed Hamed Hosseini Nasab

**Affiliations:** ^1^ Institute for Biomechanics, Department of Health Sciences and Technology, ETH Zürich, Zürich, Switzerland; ^2^ Department of Biomedical Engineering, Steadman Philippon Research Institute, Vail, CO, United States; ^3^ Julius Wolff Institute, Berlin Institute of Health at Charité-Universitätsmedizin Berlin, Berlin, Germany; ^4^ Aesculap AG, Tuttlingen, Germany; ^5^ Department of Orthopaedic and Trauma Surgery, Ludwig Maximilians University Munich, Musculoskeletal University Center Munich (MUM), Munich, Germany

**Keywords:** knee mechanics, TKA, subject-specific modelling, posterior tibial slope, soft tissue loading

## Abstract

As a solution to restore knee function and reduce pain, the demand for Total Knee Arthroplasty (TKA) has dramatically increased in recent decades. The high rates of dissatisfaction and revision makes it crucially important to understand the relationships between surgical factors and post-surgery knee performance. Tibial implant alignment in the sagittal plane (i.e., posterior tibia slope, PTS) is thought to play a key role in quadriceps muscle forces and contact conditions of the joint, but the underlying mechanisms and potential consequences are poorly understood. To address this biomechanical challenge, we developed a subject-specific musculoskeletal model based on the bone anatomy and precise implantation data provided within the CAMS-Knee datasets. Using the novel COMAK algorithm that concurrently optimizes joint kinematics, together with contact mechanics, and muscle and ligament forces, enabled highly accurate estimations of the knee joint biomechanics (RMSE <0.16 BW of joint contact force) throughout level walking and squatting. Once confirmed for accuracy, this baseline modelling framework was then used to systematically explore the influence of PTS on knee joint biomechanics. Our results indicate that PTS can greatly influence tibio-femoral translations (mainly in the anterior-posterior direction), while also suggesting an elevated risk of patellar mal-tracking and instability. Importantly, however, an increased PTS was found to reduce the maximum tibio-femoral contact force and improve efficiency of the quadriceps muscles, while also reducing the patellofemoral contact force (by approximately 1.5% for each additional degree of PTS during walking). This study presents valuable findings regarding the impact of PTS variations on the biomechanics of the TKA joint and thereby provides potential guidance for surgically optimizing implant alignment in the sagittal plane, tailored to the implant design and the individual deficits of each patient.

## 1 Introduction

Total knee arthroplasty (TKA) is a common surgery aimed at restoring knee function in individuals experiencing progressive osteoarthritis. Currently, around 400,000 primary TKA surgeries are conducted annually in the United States alone (Varacallo et al., 2022), and it is anticipated that this number will surge to 1.26 million operations per year by 2030 (Sloan et al., 2018; Shichman et al., 2023). Unfortunately, surgery outcome dissatisfaction rates varying from 10% to 30% (Ayers et al., 2022; Ferri et al., 2023; Inui et al., 2023; Nham et al., 2023), with the greatest complaints concerning persistent pain and joint stiffness, which impede subject’s ability to engage in everyday functional activities (Choi and Ra, 2016). Importantly, poor functional and clinical outcomes greatly contribute to revision surgeries, particularly among younger individuals who have undergone TKA (with revision rates of up to 22% for patients undergoing surgery below 50 years (Stone et al., 2022)).

Implant design and implantation are thought to be *the* two primary factors governing knee functionality after TKA. In particular, implant alignment is known to play a critical role in postoperative joint instability/stiffness (Sheth et al., 2017; Oussedik et al., 2020), as well as soft tissue loading patterns (Willing and Walker, 2018; Johnston et al., 2019). Several studies have assessed changes in kinematics and kinetics of the knee during walking due to variations in implant alignment in the coronal plane. These include investigations into changes in patella kinematics (McWalter et al., 2007) as well as tibiofemoral contact location and load (Halder et al., 2012; Lerner et al., 2015; Smith et al., 2016a). On the other hand, there has been a relatively small number of biomechanical investigations exploring the role of TKA implantation parameters in other anatomical planes. Specifically, in the sagittal plane, it has long been thought that an increased posterior tibial slope (PTS) can increase the moment arm of the quadriceps muscles by shifting the tibiofemoral contact points posteriorly (Ostermeier et al., 2006a; Kang et al., 2017; Wang et al., 2020). Therefore, many surgeons try to encourage femoral rollback by increasing the posterior slope of the tibial component to enhance the range of knee flexion as well as efficacy of the knee extensor mechanism. However, the consequences of such variations on articular contact mechanics and loading of other knee structures has not yet been completely clarified.

*In-vivo* studies investigating the influence of PTS on post-TKA knee joint biomechanics have been mainly limited to those using medical imaging to quantify range of motion (Bellemans et al., 2005) or to assess knee stability (Ersin et al., 2023) during passive or loaded knee flexion (Seo et al., 2013). For example, Bellemans and co-workers (Bellemans et al., 2005) evaluated maximum knee flexion angles obtained from patients with well-functioning TKAs using video-fluoroscopy and found an average gain of 1.7° flexion for every degree of PTS. In another study, Fujimoto and co-workers (Fujimoto et al., 2013) measured the femorotibial joint gap over the arc of passive knee flexion (0°–135°) and reported a significantly greater tibiofemoral gap but an improved postoperative range of motion (ROM) for joints with higher PTS angles. However, due to radiation exposure and limitations of the measurement technique, such *in-vivo* investigations have not yet been expanded to functional activities like walking and squatting.

Some *in-vitro* cadaveric studies have also assessed the impact of PTS on knee function after TKA. For example, Ostermeier and co-workers (Ostermeier et al., 2006a) used a cadaveric rig to simulate isokinetic knee flexion-extension on seven cadaveric specimens with TKA implants. They reported an improved efficacy of the knee extensor mechanism due to increased quadriceps lever arms for TKAs with larger PTS angles. Using a similar approach, isokinetic extension of the cadaveric knees was also examined, where PTS was shown to only have a small influence on tibiofemoral contact stress, but a large effect on PCL loading, after PCL-retaining TKA (Ostermeier et al., 2006b). Their findings are, however, only partially consistent with those reported by Wang and co-workers (Wang et al., 2020) who similarly found that TKAs with larger PTS angles result in more posterior femoral translation, but reported larger articular contact areas and thereby smaller contact pressures. Such inconsistent results plausibly originate from the limited capacity of *in-vitro* cadaveric studies to accurately recreate the *in-vivo* loading conditions within the knee. Moreover, cadaveric tests are inefficient for parametric investigations into the outcomes of varying surgical factors.

Given the limitations of *in-vivo* and *in-vitro* investigations, computational modelling provides an efficient toolset to simulate different approaches in orthopaedic surgeries and estimate their biomechanical outcomes (Hoy et al., 1990; Arnold et al., 2010; Carbone et al., 2015). Such numerical approaches therefore provide opportunities for more detailed investigations into the influence of PTS on tibiofemoral as well as patellofemoral kinematics, ligament forces, and knee contact mechanics. Traditionally, however, forward dynamic simulations have been unduly sensitive to parameter variations, while inverse dynamic formulations have been limited to the quasi-static boundary constraints dictated by motion capture data. Moreover, solving the muscle redundancy problem in the presence of contact and ligament forces has been technically challenging. The recent development of force dependent kinematic (FDK) approaches within musculoskeletal modelling environments (Brandon et al., 2017; Skipper Andersen et al., 2017) now enables the simultaneous prediction of muscle forces, ligament forces, cartilage contact pressures as well as secondary knee kinematics during dynamic activities while solving the inherent muscle redundancy problem. As such, FDK approaches, including the recently developed COMAK tool (Brandon et al., 2017; clnsmith, 2022) within OpenSim (Delp et al., 2007), allow joint kinematics to be iteratively updated in order to balance the joint loading conditions, and hence provide a predictive approach for evaluating the kinematic and kinetic effects of perturbing specific parameters such as PTS.

A sound understanding of the role of PTS requires access to highly accurate kinematics and kinetics. The CAMS-Knee datasets (Taylor et al., 2017) provide access to the subject-specific implant and bone geometries and precise implantation data to create detailed subject-specific models of the measured subjects. Moreover, the unique collection of skin-marker motion capture data, lower limb muscle EMG, 6 degree of freedom (DoF) knee kinematics reconstructed from video-fluoroscopy, and *in-vivo* measured knee contact forces (KCFs) enable comprehensive musculoskeletal simulations of functional activities as well as extensive validation of modelling predictions. Therefore, this study first aimed to validate a computational modelling framework against the CAMS-Knee datasets, which provided the basis for then understanding the influence of PTS on TKA joint biomechanics. To this aim, we have built a subject-specific musculoskeletal model with detailed knee joint structures reconstructed from patient-specific CT images. After a comprehensive validation of the joint kinematics and loading as well as muscle activation patterns, the model was used to simulate virtual implantations with different PTS angles to investigate the resultant changes in knee contact mechanics and soft tissue loading conditions.

## 2 Methods

### 2.1 CAMS-knee datasets

The CAMS-knee datasets (Taylor et al., 2017) report experimental data including kinematics and loading of the tibiofemoral joint in six TKA subjects performing five trials of multiple functional activities of daily living. In addition to the routine gait lab data (i.e., skin-marker trajectories, ground reaction forces (GRFs), and EMG), six components of the knee contact forces (KCFs) and moments were also measured *in-vivo* using instrumented knee implants (Heinlein et al., 2007). The 6 DoF tibiofemoral kinematics were reconstructed from video-fluoroscopy images to within 1° (all rotations) and 1 mm (in-plane) accuracy (Foresti, 2009) and reported in the datasets. For the current study, we used only the data available for level walking and squatting, where the level walking cycle was defined from heel strike to heel strike of the instrumented leg while the squat cycle was defined from upright standing, through deep flexion, to upright standing. In addition to the publicly available data, we had access to subject-specific CT images of the lower limb bones.

### 2.2 Subject specific lower limb model

**FIGURE 1:**
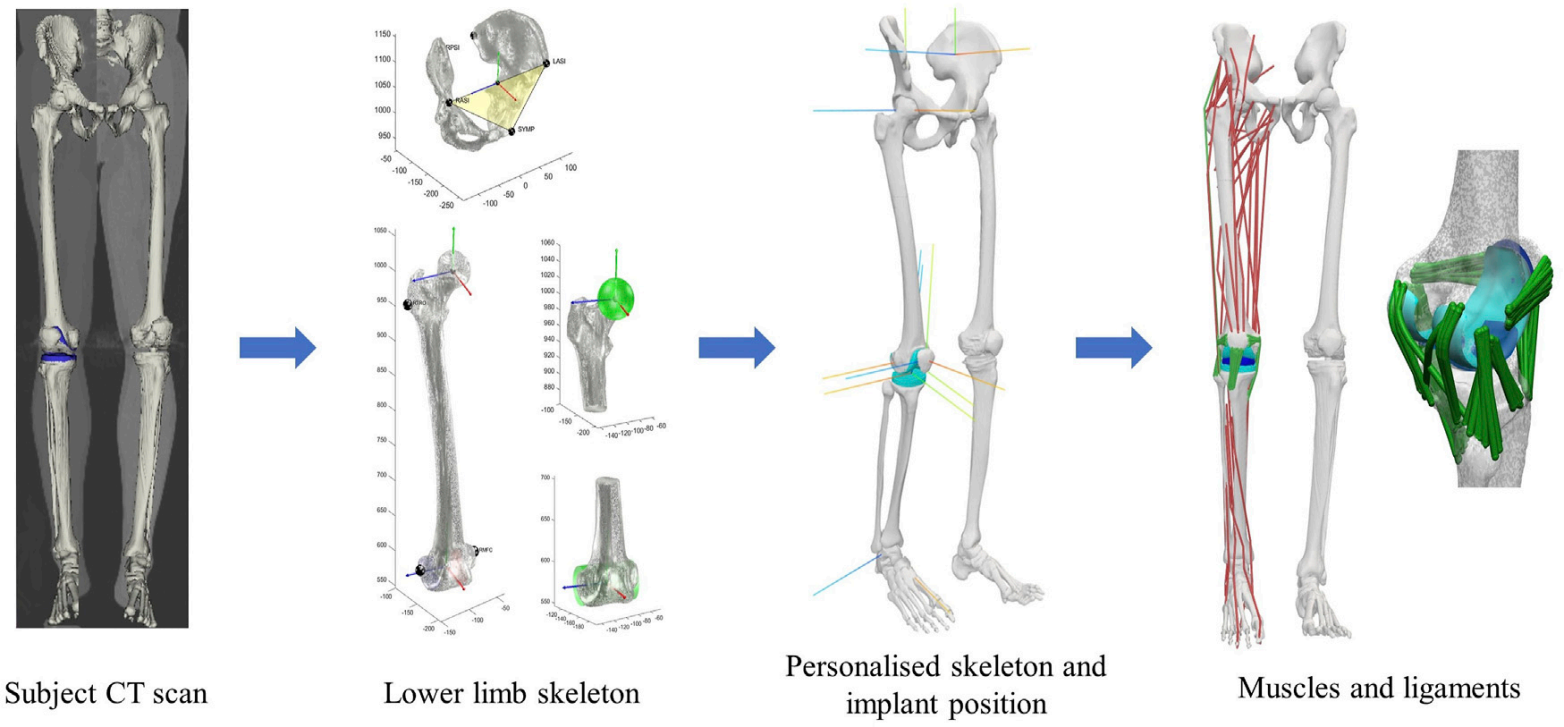
Steps towards constructing the Subject-Specific Musculoskeletal Model.

A personalized model was developed to represent subject “K5R” (65-year-old, with a height of 1.74 m and a mass of 95.6 kg) from the CAMS-Knee project. The subject underwent a cruciate-sacrificing TKA of the right knee, receiving a highly congruent implant (Innex FIXUC, Zimmer AG, Switzerland). The surgical procedure aimed to achieve mechanical alignment of the leg, leading to postoperative varus and PTS angles of 1° and 7°, respectively. For the current study, the 3D geometries of lower limb bones were reconstructed through segmentation of the subject’s CT images. To account for the exact subject-specific implantation details, implant components were accurately positioned in their parent bones to match their exact positions on the CT images. Using the STAPLE toolbox (Modenese and Renault, 2021), personalized bone and joint coordinate systems were determined and used to generate a lower limb multibody model (Figure 1). It is worth mentioning that, in this study the tibiofemoral joint was defined to allow the six DoF kinematics of the femoral component relative to the tibial component (reference frame), with the coordinate system described by Kutzner and co-workers (Kutzner et al., 2010). Here, the origin of the tibial reference frame was used to calculate and report the joint contact moments. Similarly, the patellofemoral joint was defined as a six DoF joint enabling movement of the patella relative to the femoral component (reference frame). Muscle properties and their 3D pathway parameters were scaled from a reference musculoskeletal model (Arnold et al., 2010) with 44 muscle-tendon units of the right lower limb and manually adjusted to match the subject-specific bone morphology. The optimal fibre length and tendon slack length of each muscle were optimized using the script provided by Modenese and co-workers (Modenese et al., 2016).

The tibiofemoral and patellofemoral articulating contact were modelled using an elastic foundation algorithm introduced by Lenhart and co-workers (Lenhart et al., 2015). The following soft tissue structures were then added as one-dimensional spring elements to the multibody model: superficial medical collateral ligament (sMCL), deep medical collateral ligament (dMCL), IT band (ITB), posterior capsule (pCAP), popliteofibular ligament (PFL), posterior oblique ligament (POL), lateral collateral ligament (LCL), medial patellofemoral ligament (mPFL), lateral patellofemoral ligament (lPFL), and patellar tendon (PT). The insertions of the ligaments and the number of fibre strands were decided according to average anatomical insertion sites (Meister et al., 2000; Claes et al., 2013; Alaia et al., 2014; Saigo et al., 2017; Shah et al., 2017; Dean and LaPrade, 2020). The stiffness and reference strain of each ligament bundle were initially defined based on data reported in the literature (Smith et al., 2016b). The slack length of each ligament was later optimised to maintain 2% strain at full extension of the knee.

### 2.3 Simulation of functional activities

Using the baseline subject-specific MS model, five trials each of level walking and squatting were simulated (cycle duration and maximum knee flexion angle are reported in Supplementary Table S1). Skin marker trajectories and GRF data were used as inputs to the OpenSim COMAK algorithm, which has been described in details in previous publications (Brandon et al., 2017; clnsmith, 2022). The algorithm utilizes the inverse kinematic approach (Lu and O Connor, 1999) to calculate coordinates, speeds, and accelerations of the primary joint rotations (e.g., knee flexion, hip flexion, hip adduction, … ) from the measured marker trajectories. Subsequently, a numerical optimization enables simultaneous optimization of the secondary kinematics (e.g., adduction and axial rotation of the tibiofemoral joint), muscle, ligament, and articular contact forces. The optimized solution should generate the primary joint accelerations while minimizing a cost function that solves the inherent muscle redundancy problem. In the current study, for the baseline model simulations, the knee flexion/extension angle (F-E) was prescribed based on the fluoroscopically measured kinematics. The abduction/adduction (A-A), internal/external rotation (I-E), and the three translational DoFs of the tibiofemoral joint, namely, anterior/posterior (A-P), proximal/distal (P-D), and lateral/medial (L-M) translations were estimated through COMAK optimization and verified against the CAMS-Knee fluoroscopically measured joint kinematics. In addition to the muscle activations, the components of the tibio-femoral contact force (TFCF: F_Lateral P-D_, F_Medial P-D_, F_Total P-D_, F_Total L-M_, and F_Total A-P_), as well as the three components of the contact moment (M_F-E_, M_A-A_, and M_I-E_) were predicted using the baseline musculoskeletal model and verified against EMG and the *in-vivo* forces measured by the instrumented implant.

### 2.4 Simulations with varying posterior tibial slopes

Sagittal plane alignment of the tibial component was then perturbed ±12° around the K5R 7° baseline implantation (i.e., from −5° to 19°) with 2° intervals to simulate multiple implantation scenarios with varying PTS. It is worth noting that although negative PTS values are not frequently encountered in TKA, we chose to investigate them to gain a broader understanding of potential associations between PTS and joint kinematic and kinetic parameters. Using the perturbed models, the COMAK algorithm was employed to resolve the level walking and squat simulations, as outlined in previous descriptions for the baseline model simulations. However, to enable the simulation of models with varying PTSs using the same marker trajectories obtained from subject measurements in the laboratory, the knee flexion angle was permitted to be controlled by the skin markers. The knee joint kinematics (femoral relative to tibial component, and patella relative to femoral component), kinetics, and pressure distributions within the tibiofemoral and patellofemoral joints, as well as muscle and ligament forces were extracted from the simulation outcomes and compared across the different PTSs. To assess the influence of variation in PTS on the knee joint mechanics, Spearman correlation coefficients were calculated between PTS and the peaks and ranges of different kinematic and kinetic parameters for the two activities. In a final step, we aimed to understand whether the PTS variations observed within the *in-vivo* data of the six CAMS-Knee subjects (PTS range from 5.0° to 11.0°) could be explained using the relationships revealed using our modelling framework.

### 2.5 Validation and statistical analysis

To validate the accuracy of the baseline model, we conducted comparisons between *in-vivo* measurements and *in silico* estimates. This involved using the Root Mean Square Error (RMSE) to gauge the average disparity between the model’s 6 DoF tibiofemoral kinematics and those acquired through video fluoroscopy. Additionally, RMSE was employed to quantify errors in contact force simulations by contrasting the model’s predictions with the six components of contact force and moments obtained from the instrumented knee prosthesis. Furthermore, the muscle activation patterns of lower limb muscles, as predicted by the baseline model, underwent validation against subject-specific EMG data found in the CAMS-Knee datasets.

Spearman correlation analyses were performed to assess sensitivity of the knee joint biomechanics to variation of the PTS and to identify possible relationships between PTS and *in silico* (quantified by coefficient of correlation, r_s_) estimated knee kinematic and kinetic parameters. We then explored the *in-vivo* datasets from the six CAMS-Knee subjects to find out whether relationships were also evident in the experimental data (quantified by coefficient of correlation, r_i_), and whether these match the simulation findings.

## 3 Results

### 3.1 Subject-specific model outcomes and validation: knee kinematics

**FIGURE 2:**
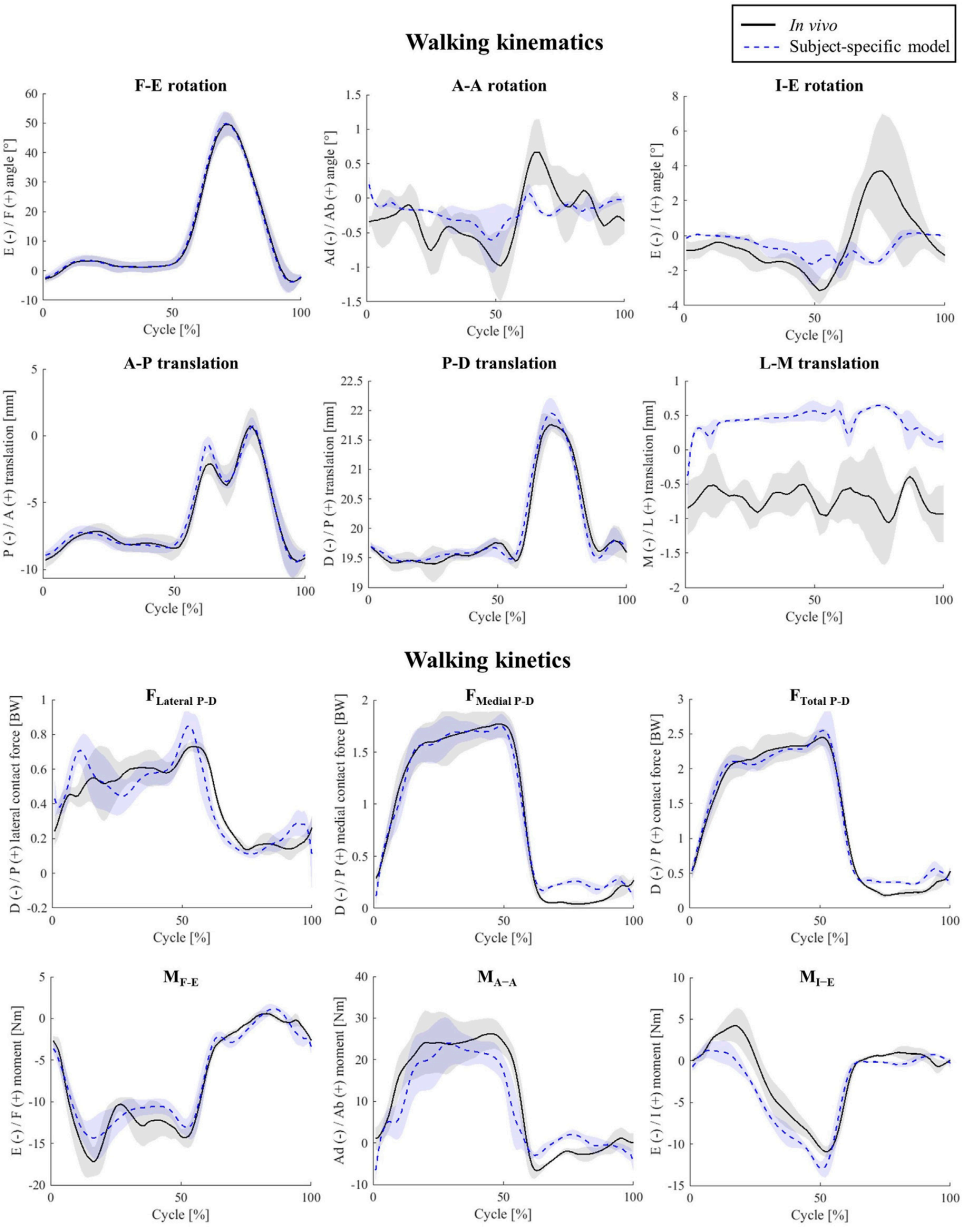
Tibio-femoral kinematics and kinetics during walking. Shaded areas show ± 1 standard deviation of the five activity trials.

For level walking simulations, the estimated tibiofemoral kinematics qualitatively matched the fluoroscopically assessed *in-vivo* joint movement patterns extremely well (Figure 2; Supplementary Figure S1), with AP translation RMSEs of below 0.3 mm (peak error of 1.5 mm), A-A and I-E rotations RMSEs of 0.3° and 1.4° respectively.

**FIGURE 3:**
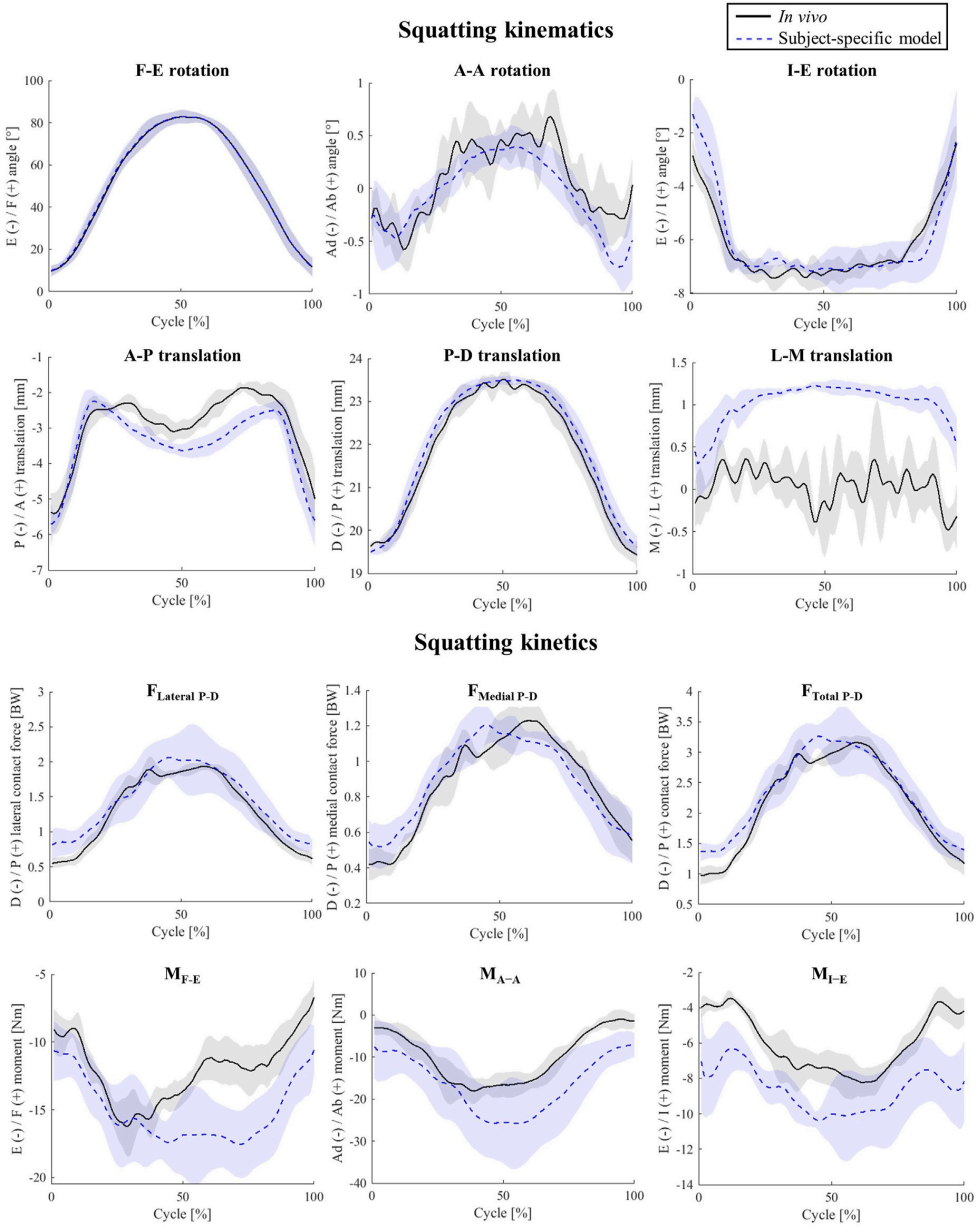
Tibio-femoral kinematics and kinetics during squatting. Shaded areas show ± 1 standard deviation of the five activity trials.

For the simulated squat activity, the model kinematics predicted the *in-vivo* joint movement patterns with only minor discrepancies. Here, the RMSEs for the A-A and I-E rotations were 0.2° and 0.4°, respectively (Figure 3). The pattern and magnitude of the A-P translation were also very accurately predicted (1.1 mm peak error).

### 3.2 Subject-specific model outcomes and validation: knee kinetics

The estimated TFCF components for level walking were also in good agreement with those measured using the instrumented knee implant (Figure 2; Supplementary Figure S1). In fact, the peak error between the simulation and *in-vivo* data was less than 10% BW for F_Total P-D_ and less than 3.8 Nm for each of the three moment components. However, there was a slight over prediction of the maximum F_Lateral P-D_ and maximum M_I-E_ (0.08 BW, and 2.05 Nm, Figure 2).

With less than 0.16 BW RMSE, the TFCF estimates for the squat trials performed by the subject nicely matched the *in-vivo* data, whereas the three joint moments were slightly over predicted, showing RMSEs ranging from 2.66 to 6.46 Nm (Supplementary Figure S1).

### 3.3 Subject-specific model outcomes and validation: lower-limb muscle activations

The baseline model was generally able to predict muscle recruitment patterns recorded by EMG sensors over the five level walking cycles measured within the CAMS-Knee datasets (Supplementary Figure S2). In particular, while activation patterns of vasti muscles, tibialis anterior, and medial hamstrings were nicely predicted by the modelling framework, the lateral hamstrings, and rectus femoris activations did not precisely match the corresponding EMG data. For the simulated squat trials, except for the hamstrings, activation patterns of other muscles were in a good agreement with their corresponding EMG signals (Supplementary Figure S3).

### 3.4 Simulation of PTS: walking

**FIGURE 4:**
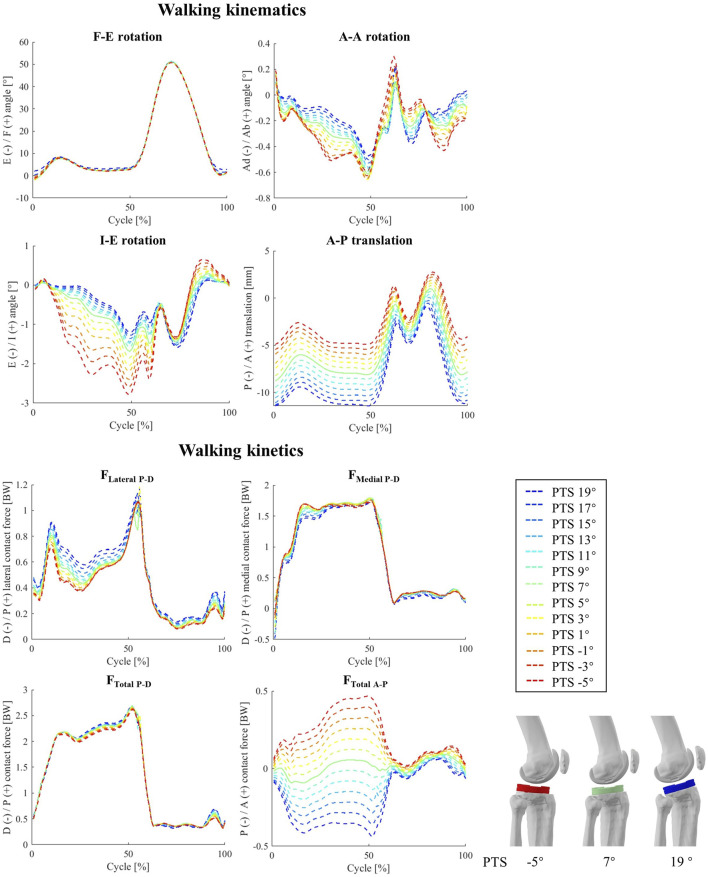
The impact of different PTS angles on tibiofemoral joint kinematics and kinetics during walking. Note: The shift in PTS was subtracted from the implant flexion to solely capture alterations in the knee flexion angle.

**FIGURE 5:**
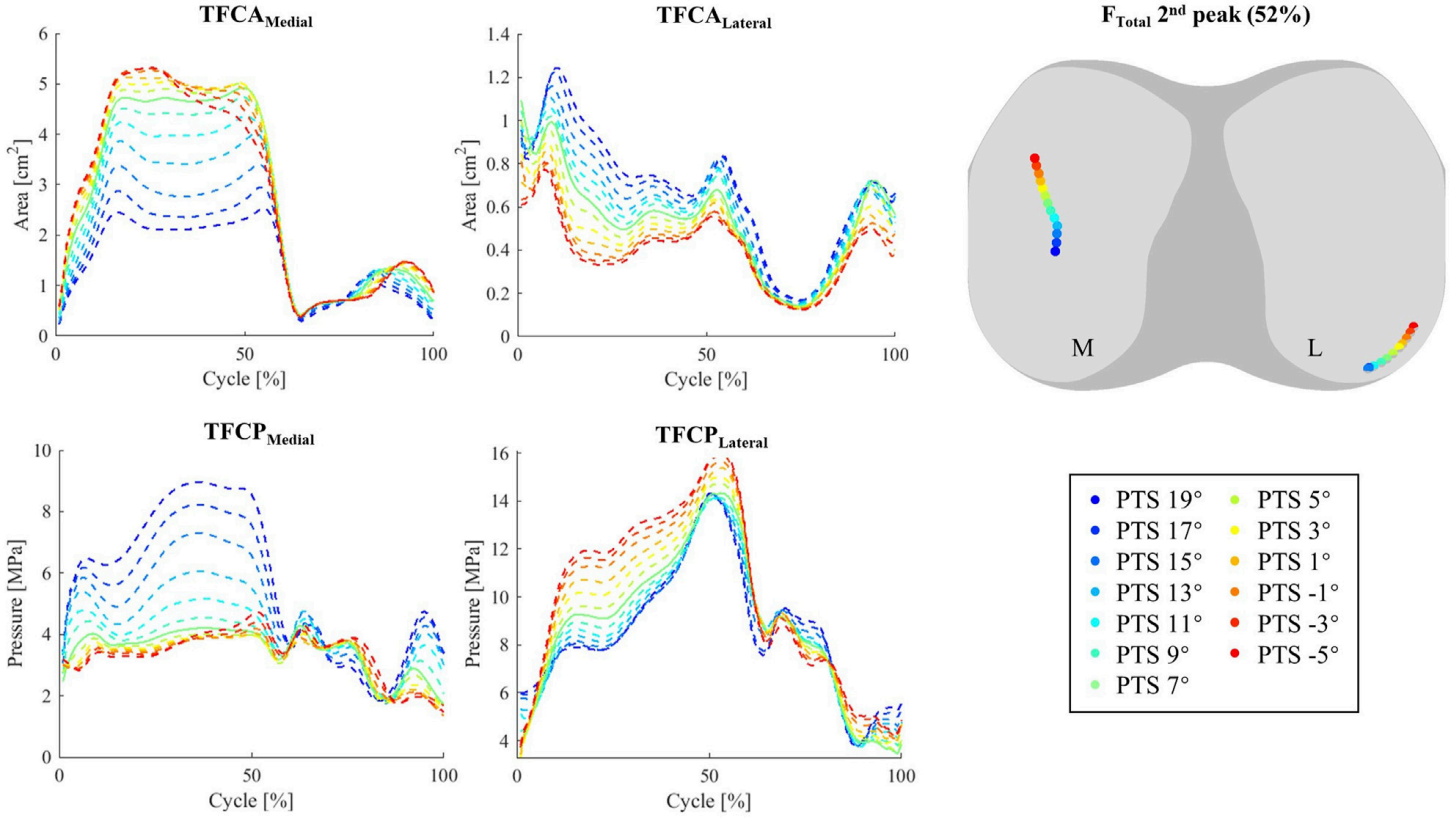
Tibiofemoral contact area (TFCA), and tibiofemoral mean contact pressure (TFCP) during walking (left). Centre of Pressure (CoP) at second F_Total_ peak (52% cycle) (right).

Increasing the PTS resulted in no significant change in the knee flexion angle during walking (Figure 4) The A-A and I-E rotation angles were found to be sensitive to PTS variations, however the magnitude of these changes was small (less than 1.6° extra internal rotation for a 24° increase of PTS, Figure 4; Supplementary Figure S4). The most visible impact of PTS on the knee kinematic parameters was observed in the relative positioning of the femoral condyles on the tibial component, where greater PTSs resulted in larger posterior femoral displacement. This was also confirmed by the more posterior centre of pressures (CoPs) on the polyethylene inlay, which was consistent across different flexion angles (Figure 5). Regarding the patellofemoral joint, it is important to note that all kinematic parameters were affected by the PTS. In particular, an increased PTS resulted in smaller patellar flexion and rotation angles, as well as in more anterior, proximal, and lateral translation of the patella (Supplementary Figure S5).

Altering the PTS significantly changed total TFCF in anterior posterior direction (r_s_ = 0.99) but did not result in significant changes in TFCF or its medial and lateral components (Figure 4). Moreover, substantial variations in tibiofemoral contact moments (−0.90<r_s_ < −1.00 for M_F-E_, M_A-A_, and M_I-E_, Supplementary Figure S4) were observed across models with different PTSs (Supplementary Figure S6). Notably, the analysis of *in-vivo* knee contact moments exhibited a strong correlation between the M_I-E_ and PTS (r_i_ = −0.71), while M_F-E_ and M_A-A_ exhibited moderate (r_i_ = -0.44) and weak (r_i_ = -0.26) correlations (Supplementary Figure S4). Additionally, an increase in PTS was associated with a reduction in the compressive and medio-lateral components of the contact force experienced by the patellofemoral joint (Supplementary Figure S7).

While shifting the medial and lateral CoPs to more posterior and central locations (Figure 5; Supplementary Figure S5). For the INNEX implant studied here, an increased PTS also reduced the tibiofemoral contact area (TFCA) on the medial side of the tibiofemoral (TF) joint (Figure 5), resulting in increased TF contact pressures (TFCPs). Opposite patterns were observed for the TFCAs and TFCPs on the lateral side. While models with larger PTSs experienced lower PF contact pressures (PFCPs, Supplementary Figure S8), no major changes in CoP translation or contact area of the patellofemoral (PF) joint were observed when varying PTS (Supplementary Figures S8, S9).

**FIGURE 6:**
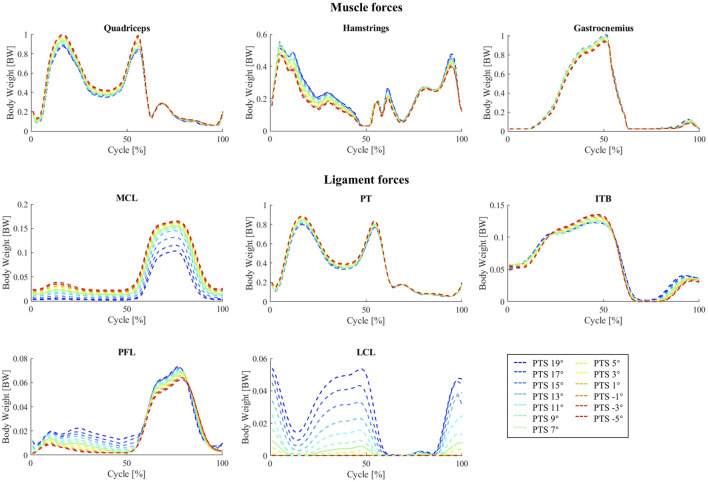
Variation of muscle and ligament forces with different PTSs during walking.

Increasing the PTS decreased the quadriceps muscle forces and produced a small increase in hamstring and gastrocnemius muscle forces (Figure 6). However, no significant changes were observed in activation patterns of other lower-limb muscles. The maximum forces experienced by the MCL (mainly the deep fibres), patellar tendon (PT), and IT band (ITB) were smaller for larger PTSs, while the PFL and LCL force peaks showed small increases (Figure 6).

### 3.5 Simulation of PTS: squatting

**FIGURE 7:**
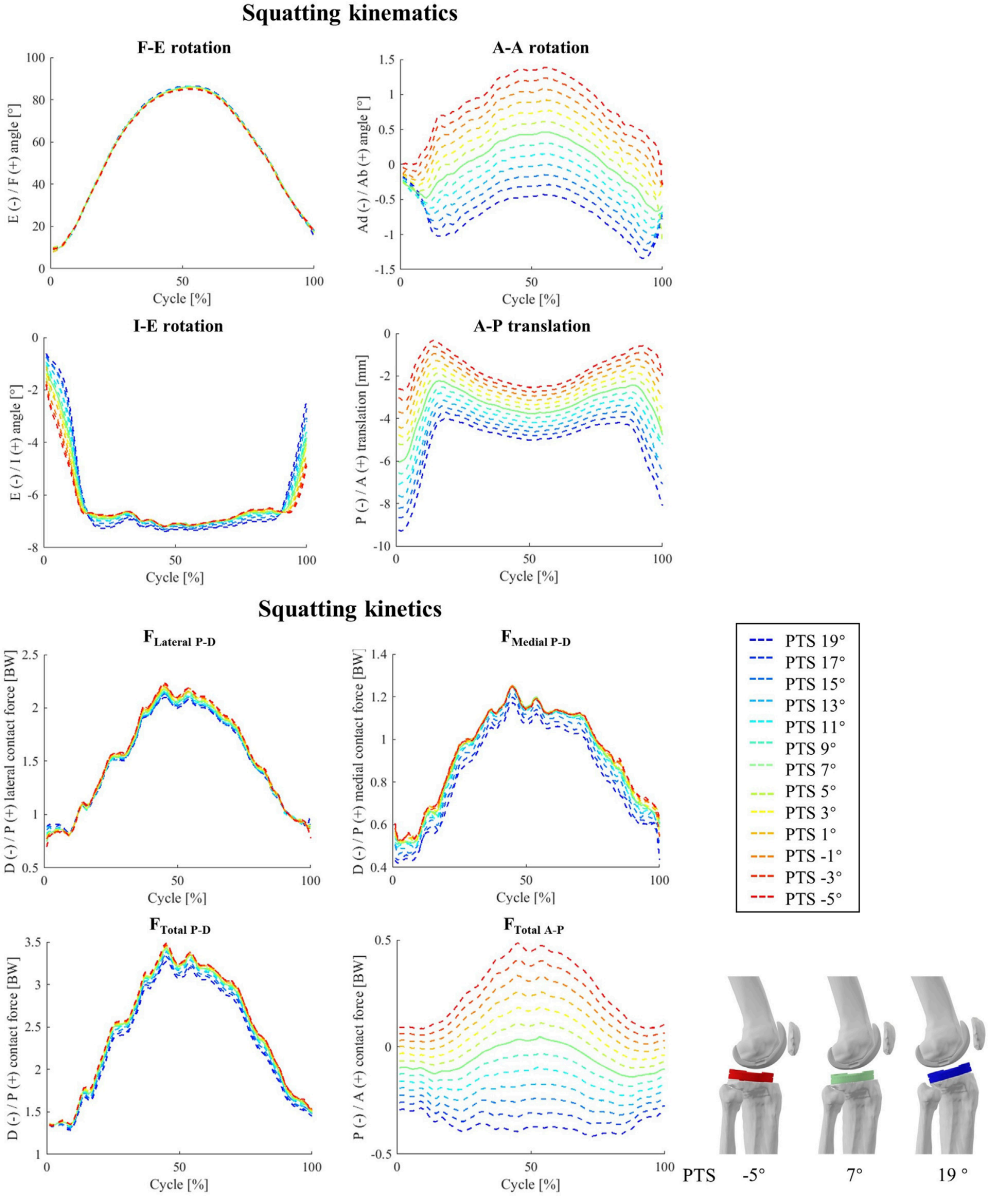
The impact of different PTS angles on tibiofemoral joint kinematics and kinetics during squatting. Note: The shift in PTS was subtracted from the implant flexion to solely capture alterations in the knee flexion angle.

For the simulated squat activity, our results indicate a strong impact of PTS on the tibiofemoral abduction angles, while such relationships were less clear *in-vivo* (−0.09<r_i_ < −0.35, Figure 7; Supplementary Figure S10). The impact of PTS on peak I-E rotation was confirmed by strong correlations observed in both the simulation and experimental data, even though the resultant changes were small (less than 0.18° variation in I-E due to 24° PTS change, Figure 7; Supplementary Figure S10). Similar to level walking simulations, an increased PTS resulted in more posterior translation of the femoral condyles relative to the tibial component, with the largest impact at the beginning and end of the squat cycle where the knee is extended. Throughout the squatting cycle, an increased PTS resulted in slight extension and medial rotation of the patella, while the impact of PTS on patellar tilt and medio-lateral translation was only observed at the beginning of the cycle (Supplementary Figure S11).

In general, for our squat simulations, an increase of PTS reduced peaks of F_Lateral P-D_, F_Medial P-D_, and F_Total P-D_ (−0.80<r_s_ < −1.00, Figure 7; Supplementary Figure S10) and resulted in larger anterior contact forces, although these changes were not evident in the experimental data (−0.53<r_i_<0.18). Moreover, clear changes were observed in M_F-E_ and M_I-E_ in response to variations in the model PTS (r_s_ = −1 for F-E and r_s_ = 1 for I-E moment, Supplementary Figure S6), but such correlations were weaker *in-vivo* (0.26<r_i_<0.44). Regarding the patellofemoral joint, the biggest impact of PTS variation was observed on the compressive contact force where an increase of PTS from −5° to 19° resulted in a 0.61 BW reduction of the maximum contact force, while slightly increasing the proximodistal shear force at the articular contact surface (Supplementary Figure S11).

Squat simulations indicated that both medial and lateral CoPs moved posteriorly in response to an increase of the PTS (Supplementary Figures S7, S12), and this was consistent for different flexion angles (Figure 7). Moreover, an increased PTS resulted in a slight medial translation of the CoPs, which was more highlighted on the medial side (Supplementary Figures S7, S12). Contrary to level walking simulation results, an increased PTS resulted in greater contact areas on the medial side, which consequently reduced the medial contact pressure (Supplementary Figure S12). For the PF joint, variations in PTS did not significantly affect the CoP location on the patellar button (Supplementary Figure S9). However, models with larger PTSs exhibited slightly reduced contact pressures during squat (Supplementary Figure S8).

The influence of PTS on the predicted muscle forces during squatting was very similar to that observed for the level walking simulations, with a reduction of the quadriceps muscle force (especially at larger flexion angles) and a small increase in hamstrings and gastrocnemius forces (mainly at small knee flexion angles) due to an increased PTS (Supplementary Figure S13). Changes in the MCL, PT and PFL forces in response to PTS perturbations were very similar between squatting and walking, however for the squat simulations, the ITB force showed a small increase with increasing PTS (Supplementary Figure S13).

## 4 Discussion

Clinically, the posterior tibial slope is varied during TKA implantation to allow beneficial outcomes regarding range of joint flexion and efficiency of the knee extensor mechanism. However, the role of PTS on soft tissue loading and articular contact mechanics is hardly understood. To address this biomechanical challenge, we developed a subject-specific musculoskeletal model based on the bone anatomy and precise implantation data provided within the CAMS-Knee datasets (Taylor et al., 2017). Using the novel COMAK algorithm that concurrently optimizes the joint kinematics, together with contact, muscle, and ligament forces enabled accurate estimations of the knee movement and tibiofemoral and patellofemoral contact loads during level walking and squatting. Once confirmed for accuracy, the baseline modelling framework was then used to study the influence of PTS on knee joint biomechanics. Our results indicated that PTS can greatly influence tibiofemoral translations (mainly in the A-P direction), while also affecting patellar tracking during both level walking and squatting. In addition, an increased PTS was found to improve efficiency of the quadriceps while reducing the forces experienced by the PT and the patellofemoral joint.

Our baseline model enabled accurate predictions of the tibiofemoral kinematic and kinetic parameters, as well as muscle activation patterns, for both level walking and squatting activities. To the best of our knowledge, this is the first study validating subject-specific model outcomes for two different activities against multiple force and motion parameters measured *in-vivo*. Importantly, the errors in our simulations of tibiofemoral axial contact force consistently ranked among the lowest in terms of RMSE when compared to those from similar musculoskeletal models using numerical optimization (0.1–0.16 BW in our simulations compared with the 0.1 to 1.7 BW range reported in studies by Moissenet et al. (Moissenet et al., 2017), DeMers et al. (DeMers et al., 2014), Knarr et al. (Knarr and Higginson, 2015), Thelen et al. (Thelen et al., 2014), and Imaninejad et al. (Imani et al., 2020)). It therefore seems that the application of subject-specific models, combined with advanced algorithms that account for soft-tissue balance and articulating contact conditions within the muscle optimisation process are able to greatly improve the accuracy of musculoskeletal modelling estimates. The small remaining errors most likely originate from generic model parameters such as ligament and muscle properties (Hosseini Nasab et al., 2019; Hosseini et al., 2020; Nasab et al., 2021; Zhang et al., 2021; Hosseini et al., 2022). Moreover, inaccuracy of the single-plane fluoroscopy setup used within the CAMS-Knee measurements might partially explain errors in kinematic parameters, especially at the mid-swing phase when the legs cross each other and limit the accuracy of segment registration (Acker et al., 2011).

Our sensitivity analysis investigating the impact of PTS on tibiofemoral joint kinematics has revealed that alterations in PTS predominantly impact the relative positioning of implant components along the A-P axis (Figure 4; Figure 7). Our results thus concur with previous *in-vitro* investigations reporting that a change of PTS can induce considerable changes in implant kinematics (Rodner et al., 2006; Shen et al., 2015; Wang et al., 2020). It is worth noting that since identical motion capture data were employed to guide the knee flexion angle (Figure 2; Figure 3). As such, while prior studies have theorized that an increased PTS results in a broader knee flexion range (Okazaki et al., 2014; Chambers et al., 2016; Ersin et al., 2023), our simulation methodology did not allow a direct *in silico* evaluation of this hypothesis. Nonetheless, the posterior positioning of the femoral condyles consistently observed at larger PTS values implies a reduced risk of impingement with the posterior femur. This observation therefore provides insight into the potential rationale behind the observed increased maximum knee flexion linked to higher PTS values.

This study not only extends our current understanding of the impact of PTS on tibiofemoral joint biomechanics but also broadens our insights into the potential implications of PTS alterations on patellofemoral kinematics and loading patterns. Specifically, our findings reveal that a greater PTS angle correlates with a notable increase in the range of patellar medio-lateral translation and tilt (Supplementary Figure S7, 8, 11). This phenomenon can be attributed to the fact that an elevated PTS leads to a more extended orientation of the femur relative to the tibial component, causing the patella to adopt a more superior contact position on the femoral component. In this position, congruency between the femoral implant component and the patellar button is reduced. Consequently, a TKA joint featuring a larger PTS may elevate the risk for patellar instability or dislocation, particularly at lower knee flexion angles. This conclusion aligns with the findings of Keshmiri and co-workers (Keshmiri et al., 2015), who conducted an intraoperative investigation on patellar tracking in 40 patients before and after computer navigated TKA and found that sagittal component alignment significantly influences patellar kinematics. Importantly, our computational approach extended their findings to weight-bearing functional activities, exposing the potential risk of patellar mal-tracking associated with excessive PTS values. Consequently, surgeons should be mindful of the potential for patellar mal-tracking and instability due to PTS alteration, as it may contribute to postoperative anterior knee pain, one of the most common complications following TKA (Miller et al., 2001; Kienapfel et al., 2003; Heinert et al., 2011).

As an important finding, our level walking simulations revealed no significant changes in axial TFCF due to the varied PTS (Supplementary Figure S4), while squat simulations on models with larger PTSs resulted in slightly smaller contact force peaks (Figure 7; Supplementary Figure S10). This generally concurs with findings of a previous investigation reporting no significant changes in TFCF peaks in response to PTS perturbation (Marouane et al., 2014). Our findings are also supported from a biomechanical perspective, as it is widely acknowledged that TFCFs are primarily influenced by muscle activation patterns rather than implant alignment strategies (Winby et al., 2009; Smith et al., 2016a; Frigo and Donno, 2021; Febrer-Nafría et al., 2023). Moreover, even though our level walking simulations indicate that models with higher PTSs exhibit smaller quadriceps forces (0.12 BW for 24° change in PTS, Figure 6), an increased PTS resulted in larger forces in the knee flexor muscles (Figure 6). Consequently, the overall alteration in the tibiofemoral contact force was negligible. Although small differences in the magnitudes of TFCFs do not raise significant concerns in the context of intraoperative PTS decisions, the increased F_Total A-P_, the observed posterior shift in the medial and lateral CoPs together with the increased TFCPs (Supplementary Figure S4-6, 12) do indeed raise questions about the potential negative impact of excessive PTSs on implant durability and wear patterns as has also been discussed in the literature (Wasielewski et al., 1994; Kang et al., 2018a; Pourzal et al., 2020). Therefore, it is essential to expand the scope of boundary conditions in preclinical mechanical testing (Iso, 2002; Iso, 2014) to encompass the full spectrum of potential PTSs that surgeons may consider in TKA. As far as the patellofemoral joint is concerned, we observed a meaningful decrease in the PFCFs and PFCPs with higher PTSs in both studied activities (Supplementary Figure S9). Given the relatively small variations in patellofemoral kinematics resulting from PTS alterations, it appears unlikely that an increase in PTS has a detrimental effect on patellofemoral joint loading, which has also been reported previously (Kang et al., 2018a; Kang et al., 2018b).

In general, for both movements simulated in this study, the muscle activation patterns estimated by our modelling framework closely match the corresponding EMG signals captured *in-vivo* (Supplementary Figures S1, S2) as well as those reported in other modelling investigations using the CAMS-Knee datasets (Imani et al., 2020; Wagner et al., 2022). Nonetheless, in line with numerous preceding musculoskeletal modelling investigations, our analyses predicted the peak activity of the rectus femoris muscle to occur at the midpoint of the level walking cycle, representing a noticeable deviation from the *in-vivo* EMG signals (Supplementary Figure S2). This deviation is likely attributable to the well-documented crosstalk between the vastii and rectus femoris muscles (Winter et al., 1994; Farina et al., 2002; Germer et al., 2021). The discrepancy in predicted hamstring muscle activation patterns for squat simulations primarily arises from inability of available cost functions used for muscle optimization to accurately estimate the subject-specific co-contraction between knee flexor and extensor muscles, a phenomenon corroborated in the literature (Crowninshield and Brand, 1981; Forster et al., 2004; Nguyen et al., 2019). According to our simulation results, larger PTSs were associated with decreased quadriceps muscle forces during both activities (Figure 6; Supplementary Figure S13; up to 18.4% across the modelled 24° PTS change). The enhanced efficiency of the knee extensor mechanism at higher PTSs is plausibly due to the increased posterior translation of the femoral component, leading to an increased lever arm for the quadriceps muscles. Hence, elevating the PTS may serve as a viable strategy for addressing insufficient capacity of the knee extensor musculature commonly observed in TKA patients (Fujimoto et al., 2013; Khow et al., 2022).

This study had several limitations that should be acknowledged. Firstly, certain parameters in the subject-specific model used as the baseline for assessing the influence of PTS on knee joint biomechanics were not entirely personalized, such as ligament properties and muscle pathways. However, it is important to note that the comparison against *in-vivo* joint kinematics, KCFs, and EMG patterns provides a strong foundation for the validity of our simulation pipeline. Furthermore, the within-study comparison of outcomes across different PTS values mitigates the potential impact of baseline model inaccuracies. Secondly, some of the correlations suggested by our simulations did not align with the *in-vivo* data from the CAMS-Knee datasets. This discrepancy may be attributed to numerous subject-specific variables acting as confounding factors, potentially obscuring the influence of PTS on knee joint mechanics. Specifically, the six subjects measured within the CAMS-Knee project had distinct implantation specifications, resulting in varying knee alignment parameters (Febrer-Nafría et al., 2023). This high level of diversity makes it challenging to isolate and study the specific impact of PTS on joint functionality in this particular cohort. In addition, the TFCAs and TFCPs were extremely sensitive to the implant congruency. Given that the investigated implant (Innex FIXUC implant) featured a highly congruent design, any generalization of the biomechanical interrelationships with PTS to other implant designs should be exercised with caution, especially since less constrained implants might well exhibit more extreme responses to variations in sagittal plane implantation.

To conclude, this study used one of the most comprehensive biomechanical datasets comprising knee anatomy, kinematics, and internal loading conditions to establish an *in silico* framework to investigate the influence of posterior tibial slope on the knee joint mechanics and muscle forces after total knee arthroplasty. The rigorous validation against 14 *in-vivo* kinematic and kinetic parameters as well as EMG data ensured the unique capability of the model to accurately estimate knee joint behaviour during both level walking and squatting activities. Sensitivity tests examining the role of PTS revealed significant changes in medial and lateral tibiofemoral CoP positioning, considerable variations in anterior-posterior TFCF, as well as alterations to patellofemoral translation and tilt during functional activities. Notably, higher PTSs demonstrated the potential to enhance quadriceps muscle efficiency while reducing the strain on vital structures, such as the patellar tendon and the patellofemoral interface. Simultaneously, however, excessive PTSs should be avoided due to potential issues with patellar tracking and possible instability, as well as altered contact mechanics at the tibio-femoral joint. This study offers valuable insights into the implications of PTS variations on TKA joint biomechanics. Further simulations with altered implant congruency and PTS may help to understand how different implants react to variations in PTS, thereby provide potential guidance for surgically optimizing implant alignment in the sagittal plane according to the implant concept and each patient’s specific deficits.

## Data Availability

The original contributions presented in the study are included in the article/Supplementary Material, further inquiries can be directed to the corresponding author.
