# Supplementary material for: Posterior tibial slope influences joint mechanics and soft tissue loading after total knee arthroplasty
Source: Front Bioeng Biotechnol. 2024 Apr 15;12:1352794. doi: 10.3389/fbioe.2024.1352794 (PMC11056792; doi:10.3389/fbioe.2024.1352794)
Supplement: Supplementary file 1 [file DataSheet1.PDF]

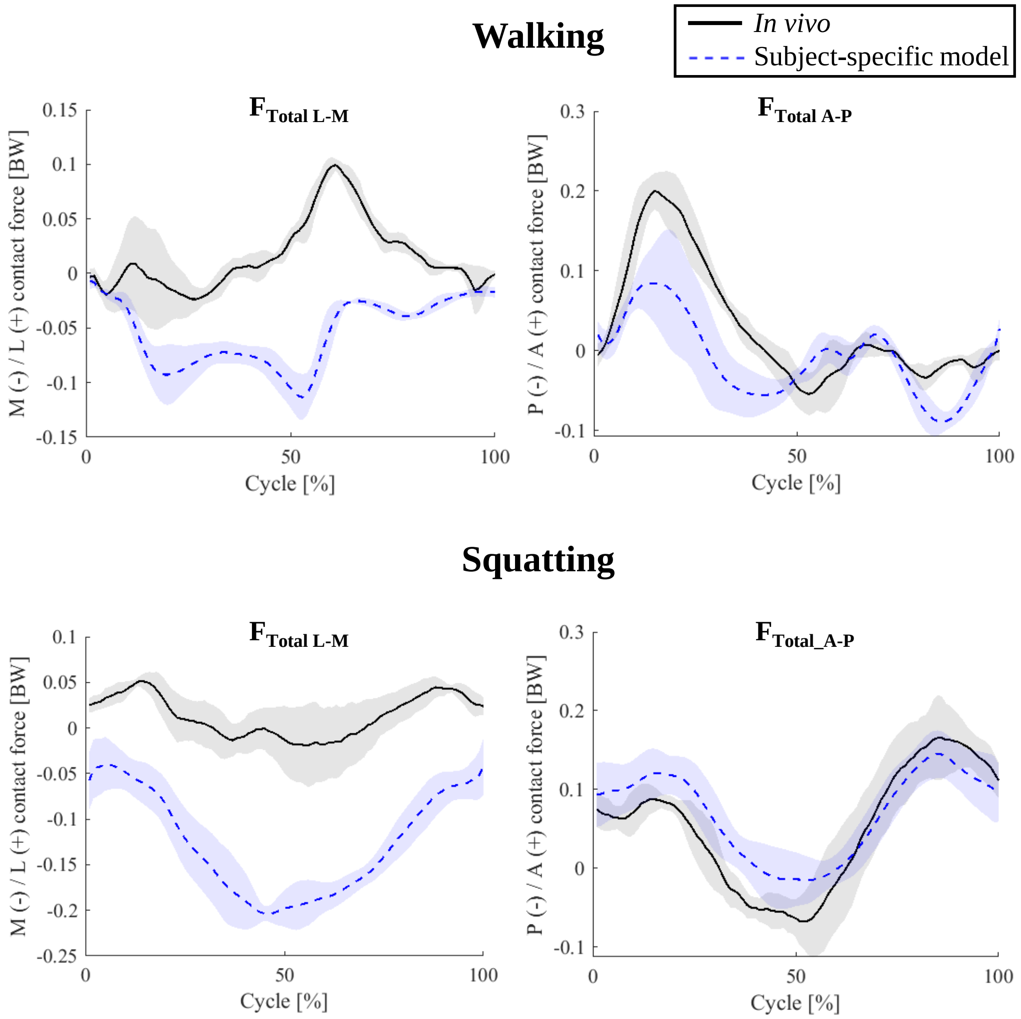

Figure S1. L-M and A-P components of the tibiofemoral contact force during walking and squatting

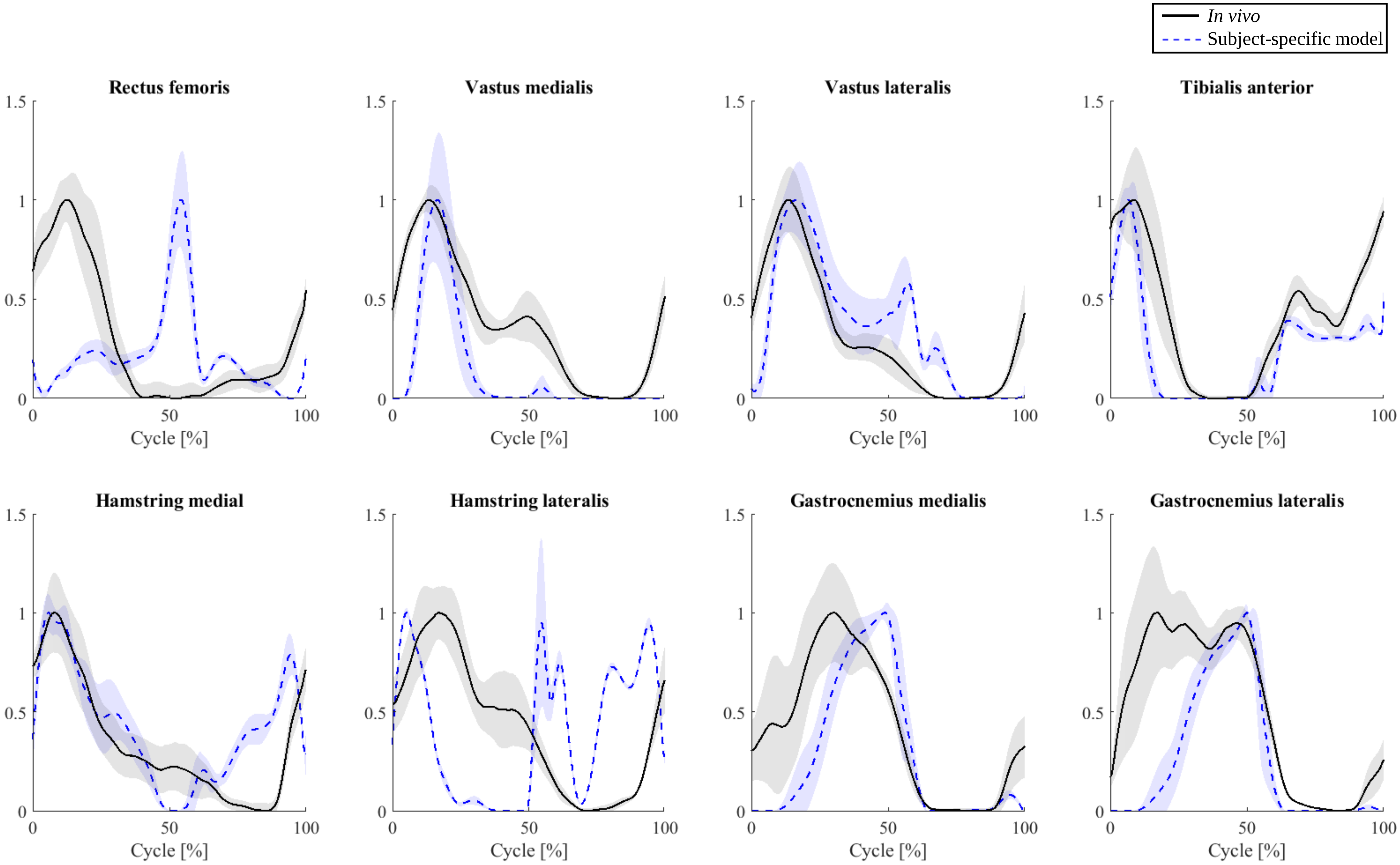

Figure S2. EMG patterns measured in vivo (in black) vs. predicted muscle activation patterns (in blue) for walking

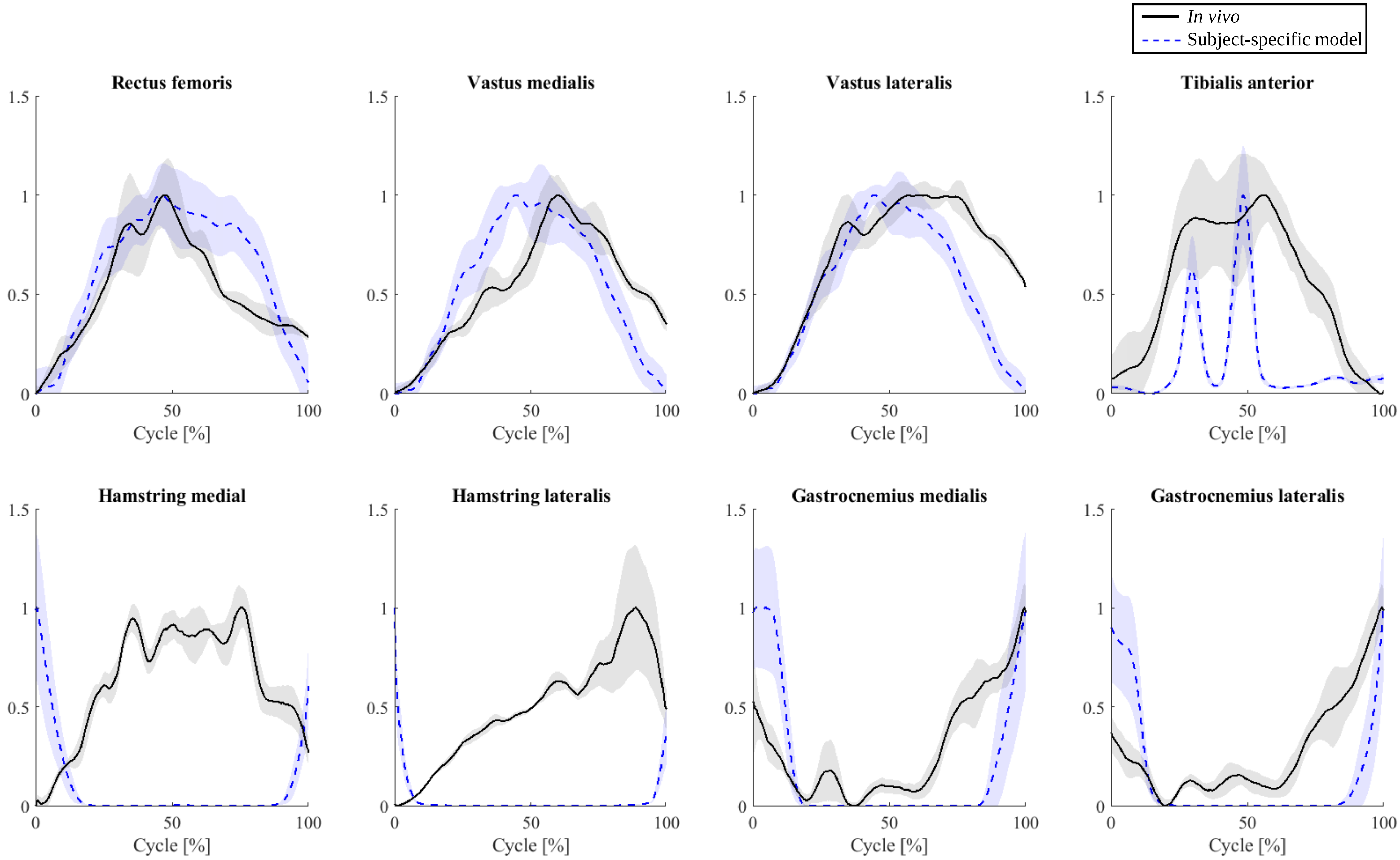

Figure S3. EMG patterns measured in vivo (in black) vs. predicted muscle activation patterns (in blue) for squatting

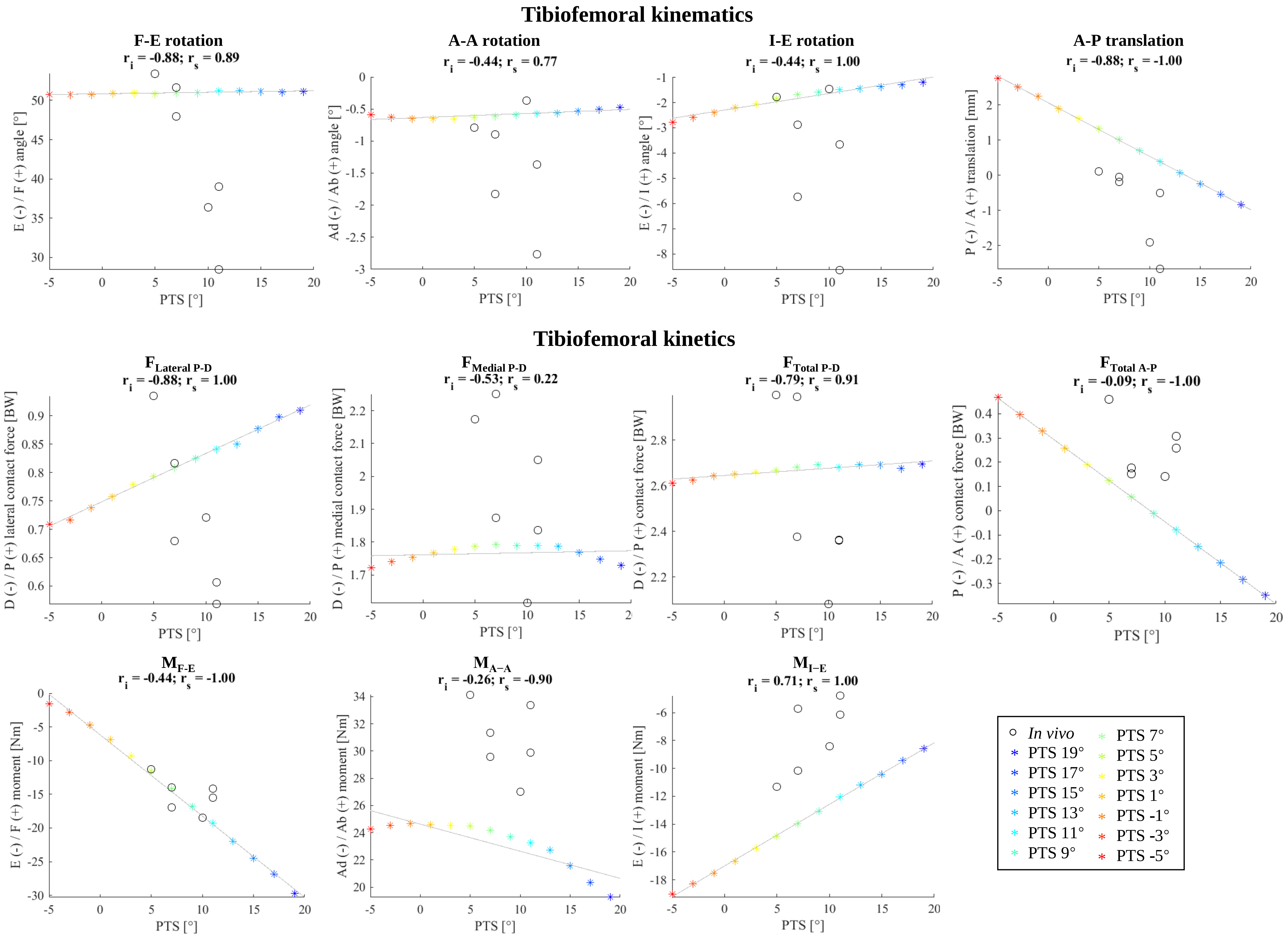

Figure S4. Correlation between PTS and tibiofemoral kinematic and kinetic parameters during walking

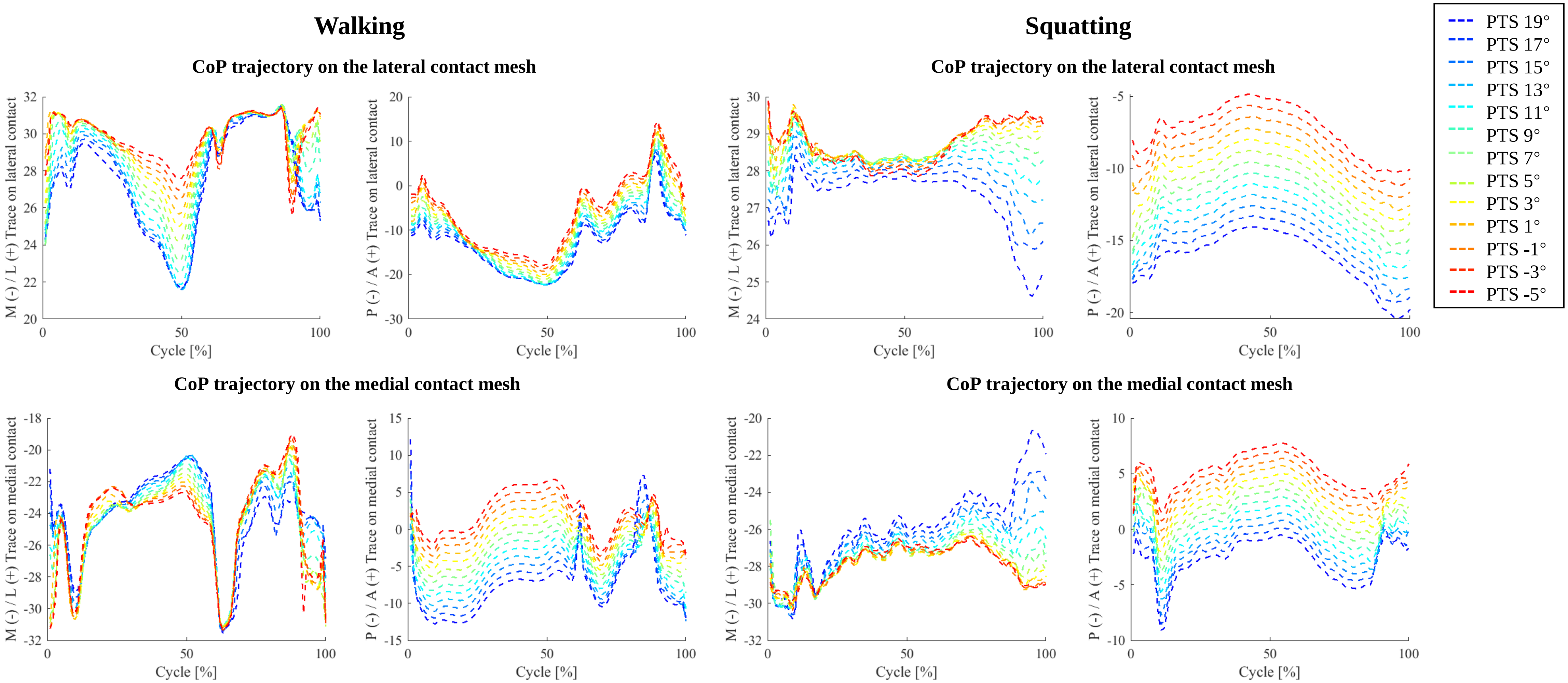

Figure S5. CoP trajectory of the tibiofemoral contact during walking and squatting for different PTS angles

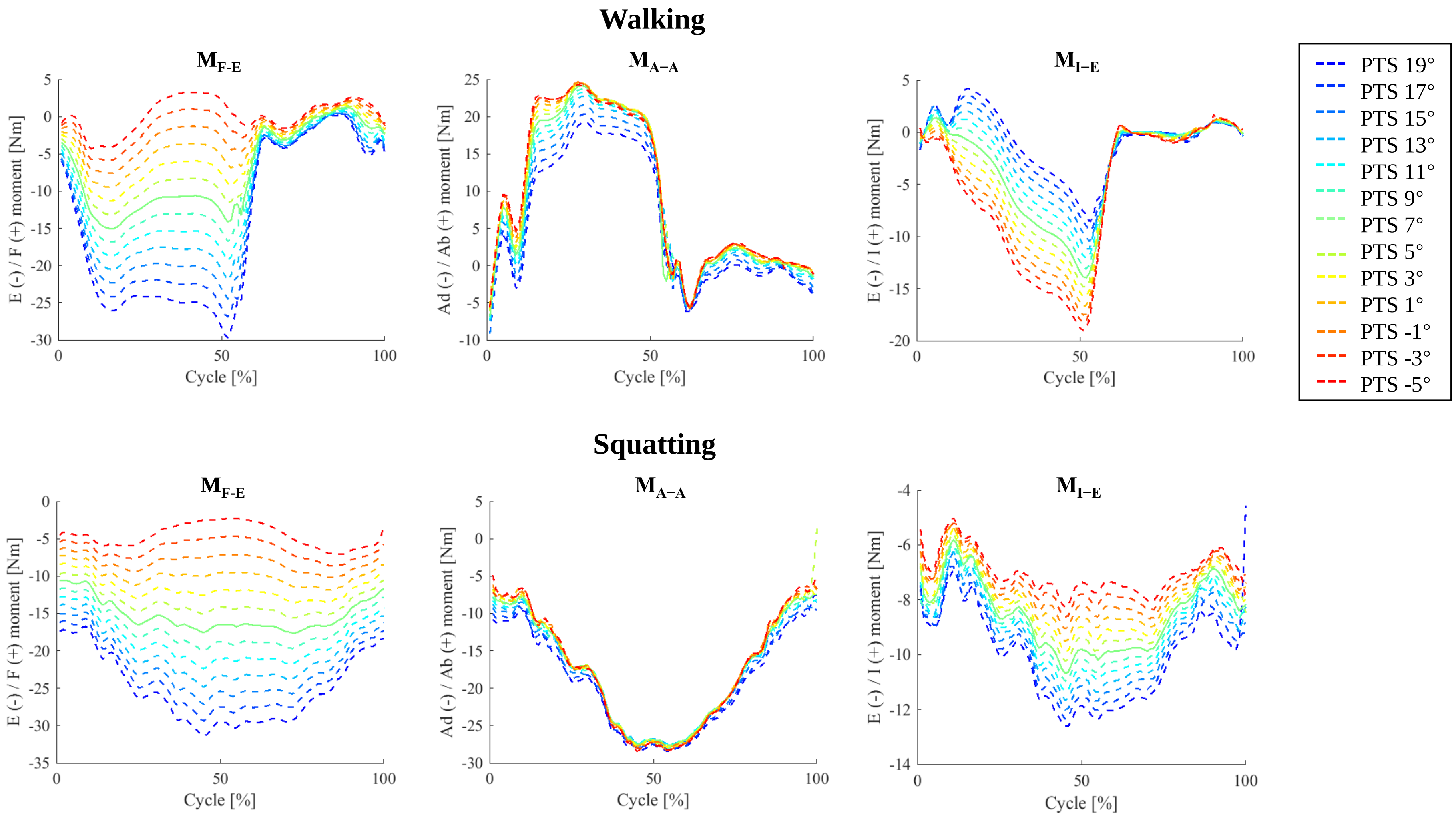

Figure S6. Tibiofemoral contact moments during walking and squatting for different PTS angles

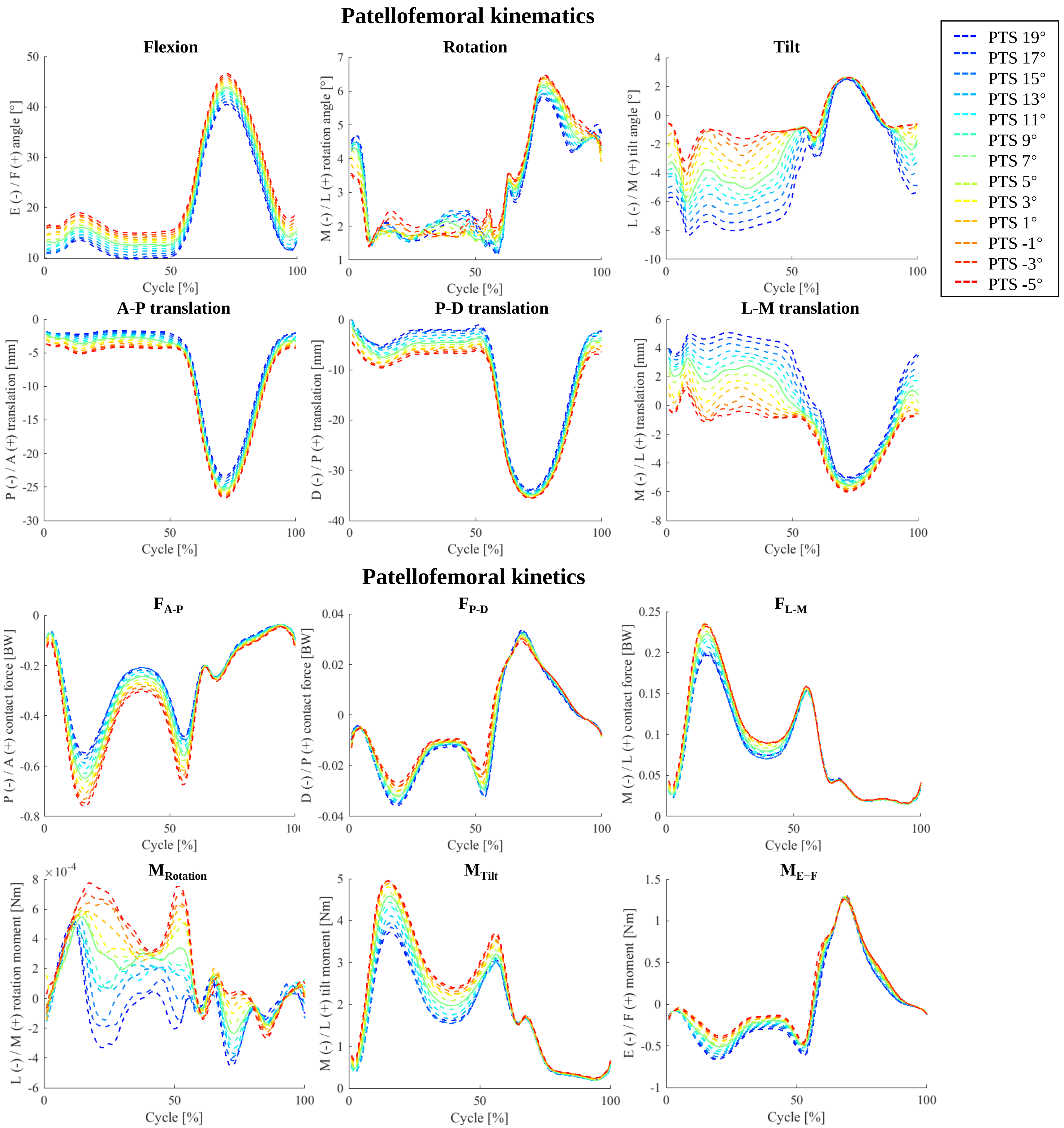

Figure S7. The impact of PTS angle on kinematics and kinetics of the patellofemoral joint during walking

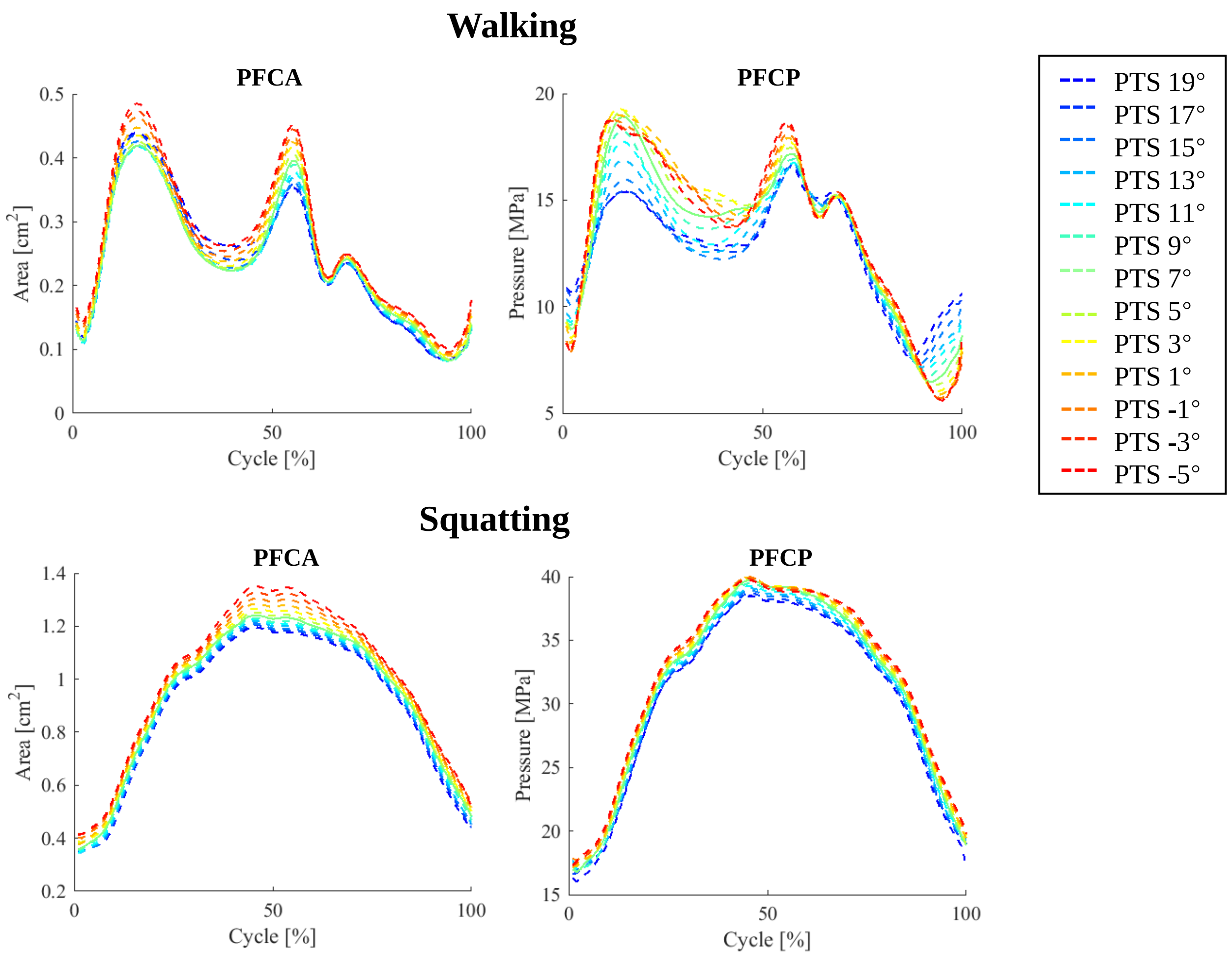

Figure S8. Patellofemoral contact area (PFCA), and patellofemoral contact pressure (PFCP) for a representative walking and squat cycle.

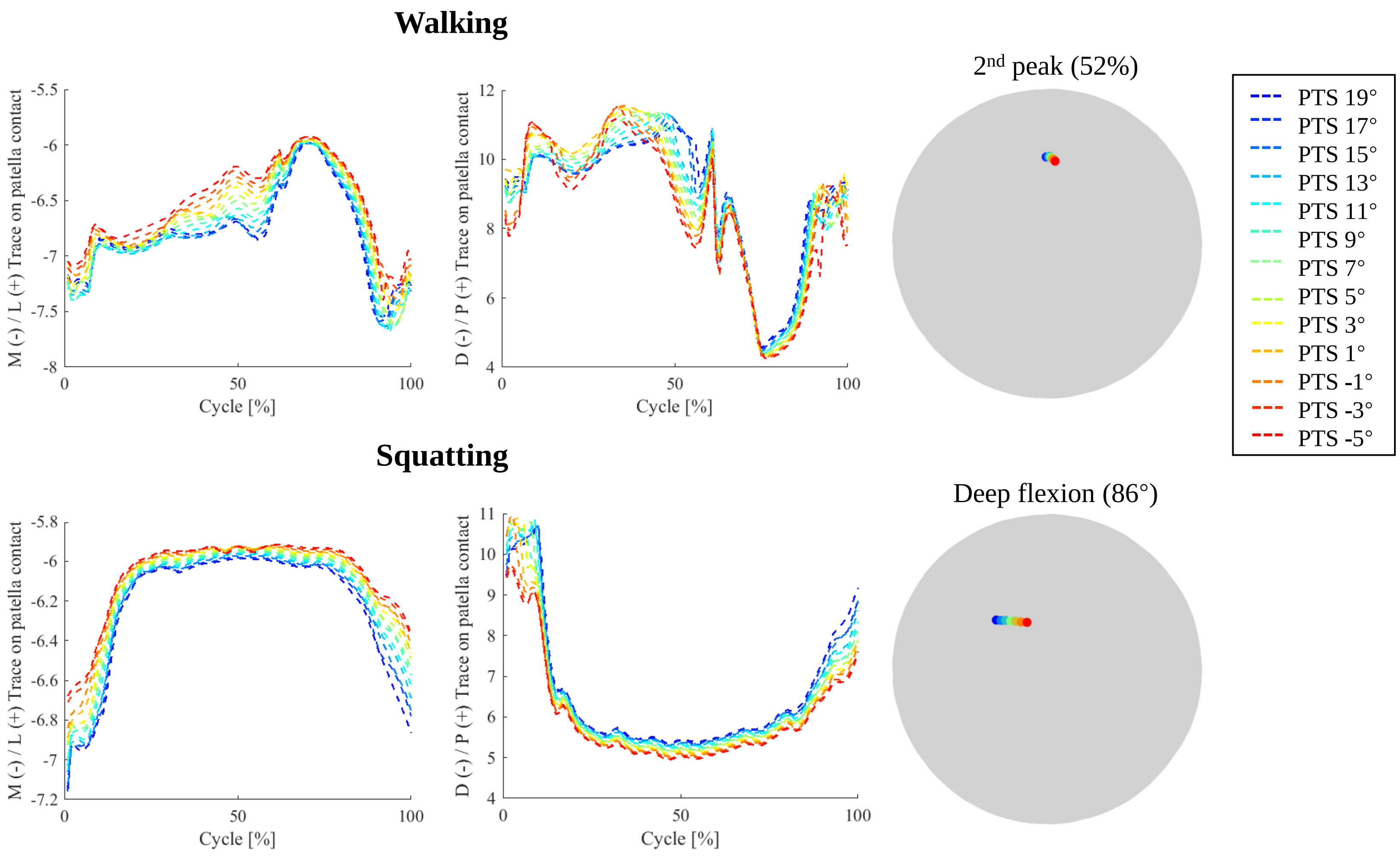

Figure S9. CoP trajectory of the patellofemoral contact during walking and squatting for different PTS angles; Centre of Pressure (CoP) at the incidence of peak tibiofemoral contact force

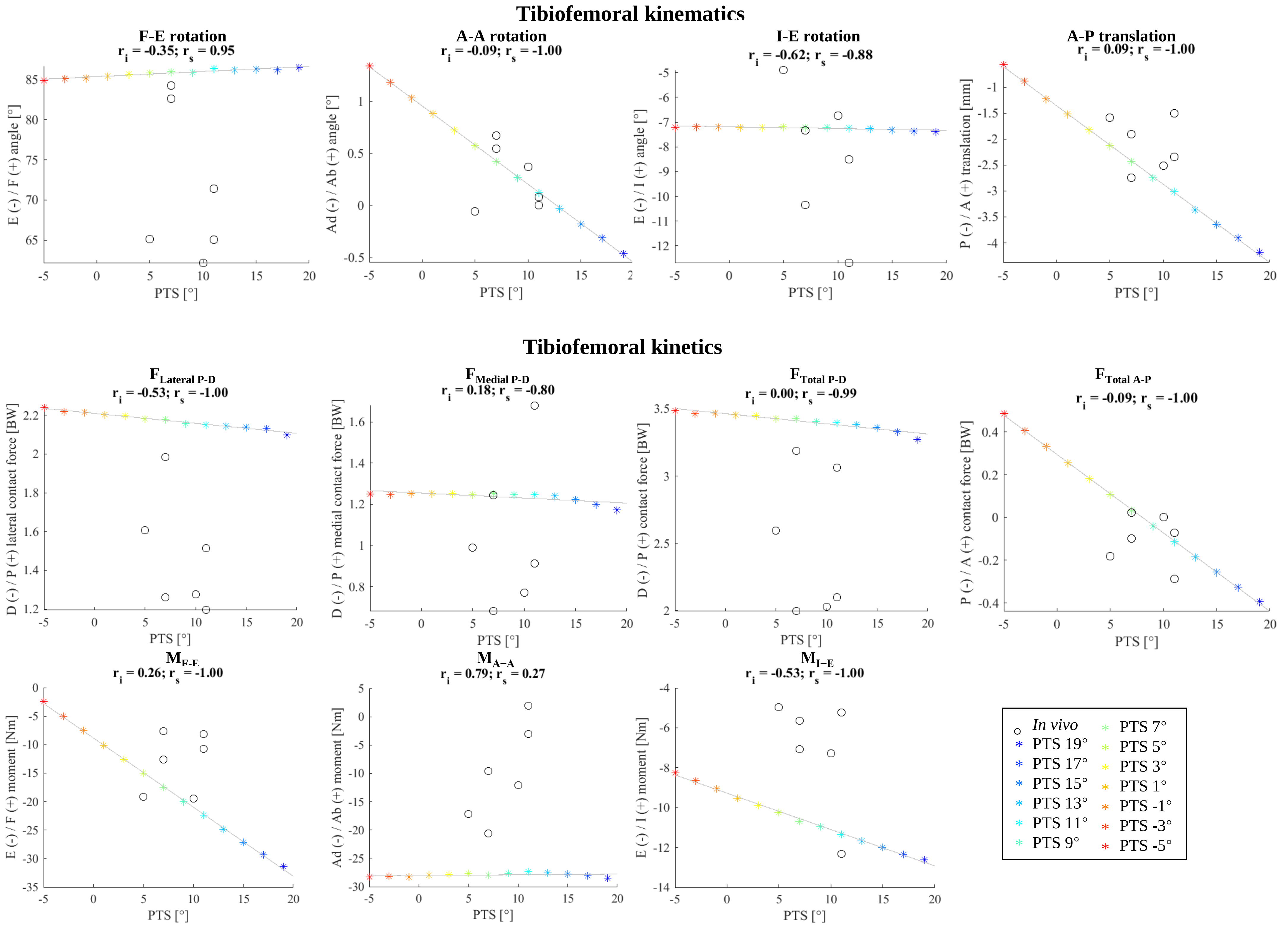

Figure S10. Correlation between PTS and tibiofemoral kinematic and kinetic parameters during squatting

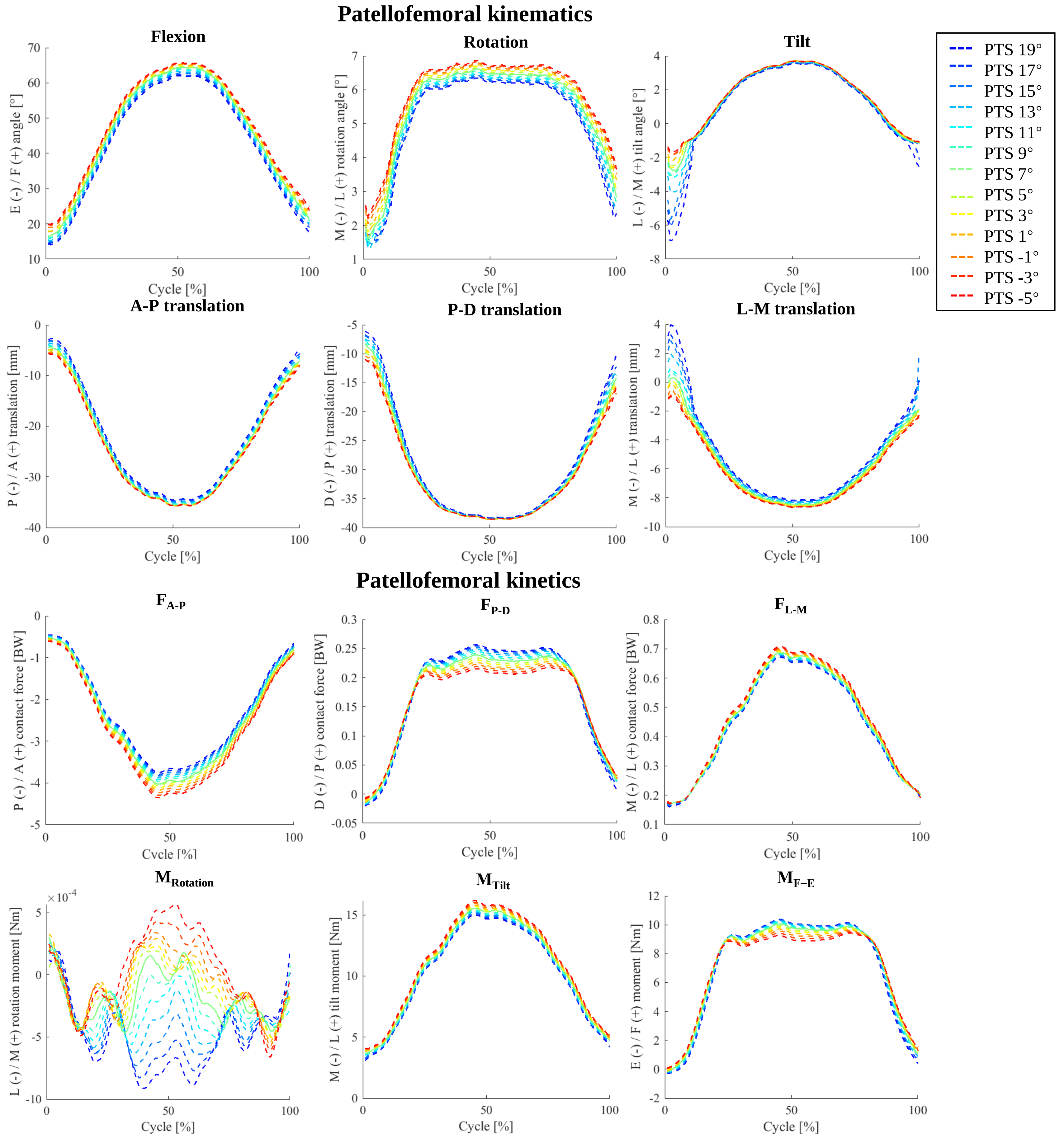

Figure S11. The impact of PTS angle on kinematics and kinetics of the patellofemoral joint during squatting

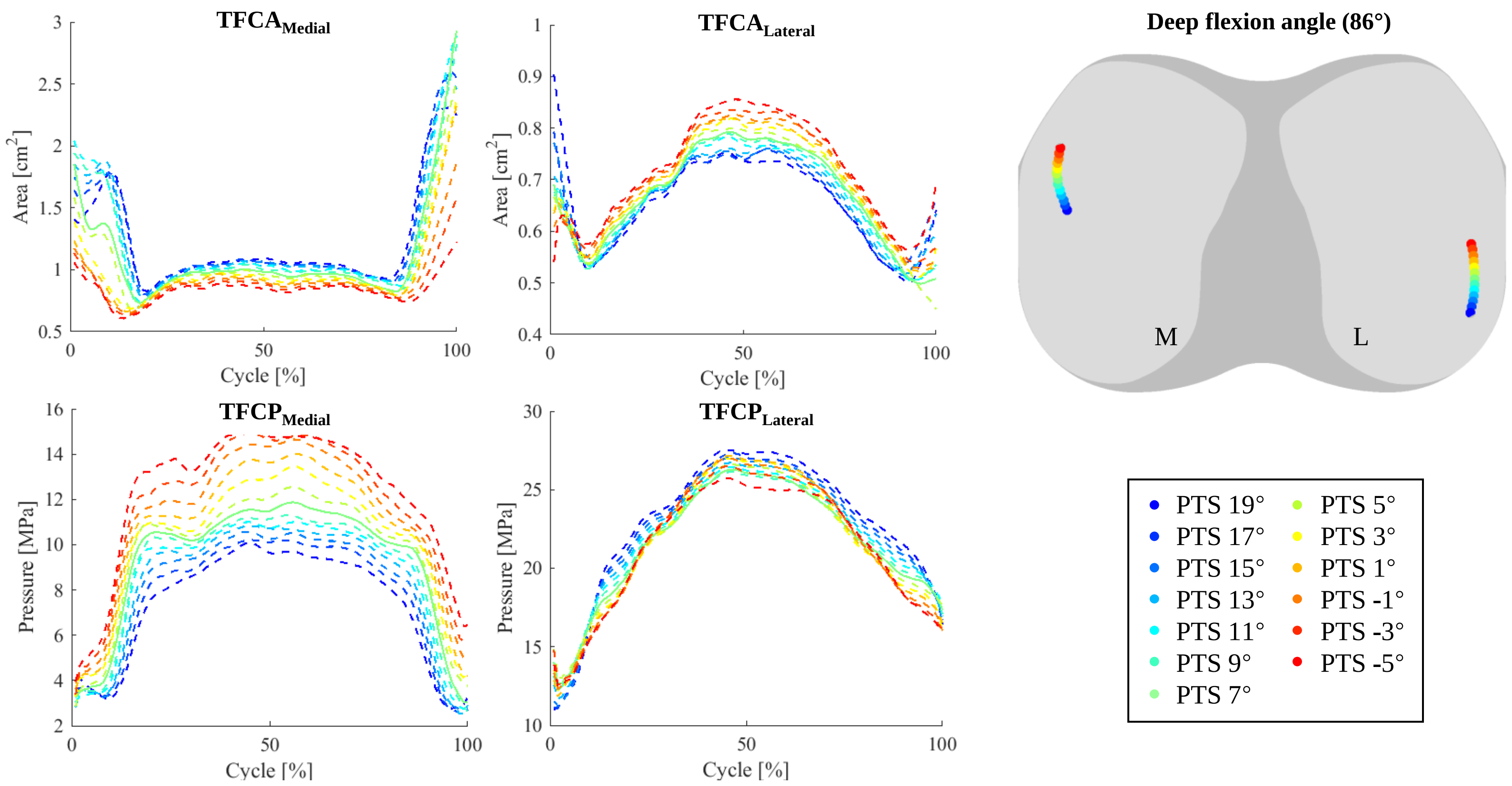

Figure S12. Tibiofemoral contact area (TFCA) and tibiofemoral average contact pressure (TFCP) for a representative squat cycle (left). Centre of Pressure (CoP) at deepest knee flexion angle during squat (right).

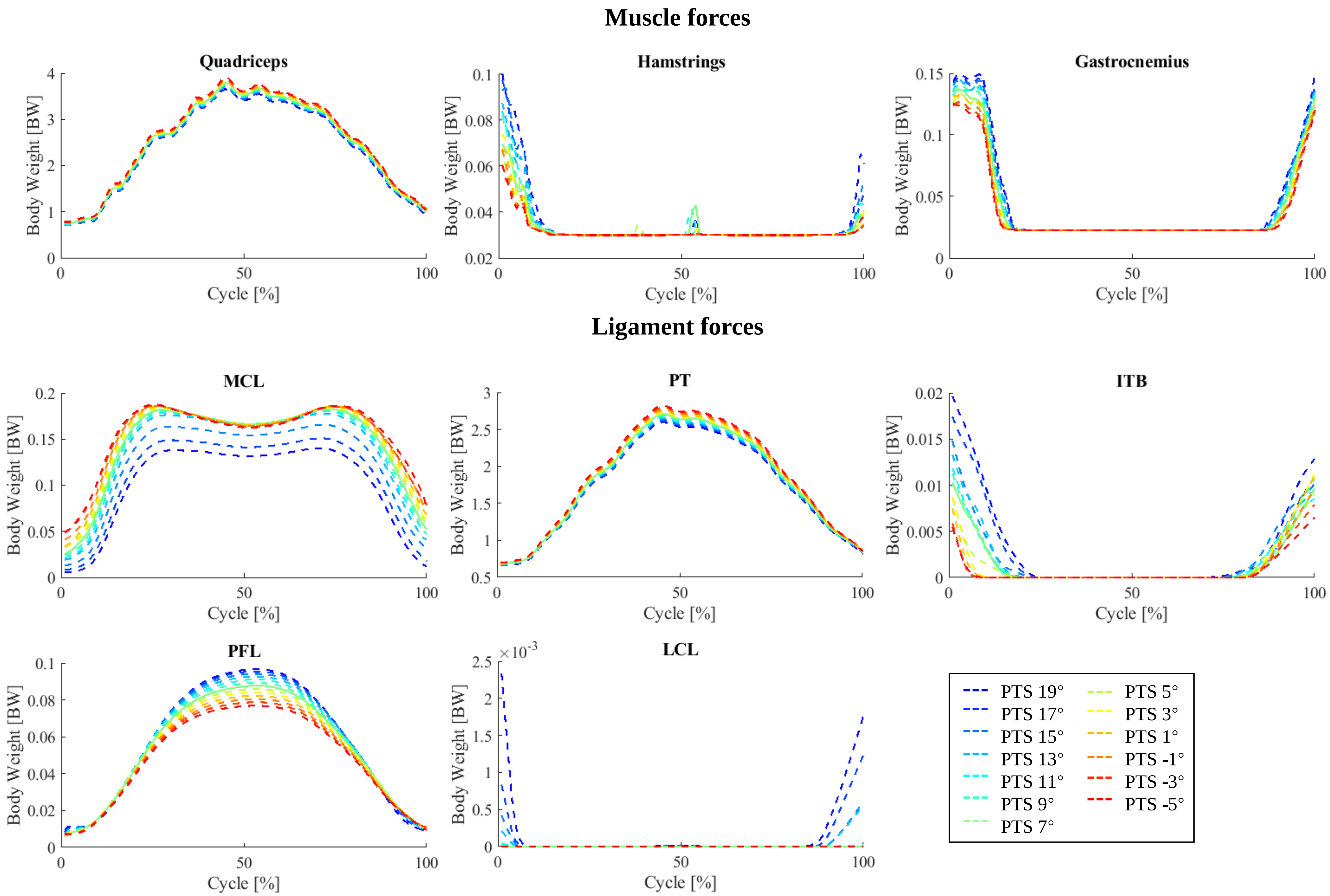

Figure S13. Muscle forces and soft tissue loading patterns during squatting for different PTSs

Table S1. Time and maximum flexion angle of the five trials of walking and squatting

		Trial 1	Trial 2	Trial 3	Trial 4	Trial 5
Level walking	Time [s]	1.48	1.56	1.32	1.48	1.48
	Maximum flexion [°]	49.4	50.4	56.1	48.3	44.5
Squatting	Time [s]	2.88	3.00	3.28	3.24	2.96
	Maximum flexion [°]	83.6	82.9	85.0	80.5	85.7
